# A Case of Cobalamin Deficiency and Macrocytic Anemia Secondary to Sunitinib

**DOI:** 10.7759/cureus.4310

**Published:** 2019-03-25

**Authors:** Jarred P Reed, Joan Chung, Natasha Banerjee

**Affiliations:** 1 Oncology, Olive View - University of California Los Angeles Medical Center, Sylmar, USA; 2 Internal Medicine, Olive View - University of California Los Angeles Medical Center, Sylmar, USA

**Keywords:** sunitinib, macrocytic anemia, cobalamin, folate, toxicity, renal cell carcinoma, tyrosine kinase inhibitor

## Abstract

Sunitinib is an oral tyrosine kinase inhibitor (TKI) commonly used in the treatment of renal cell carcinoma (RCC). Among a broad range of toxicities, anemia and macrocytosis are common in patients treated with sunitinib. Correlation between sunitinib-associated macrocytosis and cobalamin deficiency has been reported in small case series and retrospective analyses, although others have not found an association. Here, we present a case of transfusion-dependent macrocytic anemia with cobalamin and folate deficiency in a patient with RCC treated with sunitinib.

## Introduction

Sunitinib is an oral inhibitor of tyrosine kinases including vascular endothelial growth factor receptor (VEGFR), and has classically been used as a first-line treatment in advanced RCC. Due to off-target effects, the VEGFR-targeted TKIs have a number of class-specific side effects including hematologic toxicity. In a pivotal phase III trial, anemia (71%), neutropenia (72%), and thrombocytopenia (65%) were frequent events in patients treated with sunitinib for metastatic RCC [1]. Elevations in mean corpuscular volume (MCV) are also common, and in previous reports have been shown to occur in at least of patients on sunitinib after a median of 3 cycles of treatment [2]. Associations between sunitinib and deficiencies of folate and cobalamin (or vitamin B12) are less well-described but have been shown in small case series [3-5]. The case below involves a patient who developed macrocytic anemia with deficiencies of cobalamin and folate and bolsters evidence that megaloblastic anemia can occur in a subset of patients treated with sunitinib.

## Case presentation

A 71-year-old male presented with metastatic clear-cell RCC. In November 2016, he was incidentally found on imaging to have a left renal mass but declined further workup and was lost to follow-up. The patient was subsequently noted on routine laboratories in September 2017 to have creatinine elevation to 1.45 mg/dL from an unknown baseline. Renal ultrasound revealed a solid left kidney mass measuring up to 10 cm. Systemic imaging with computed tomography of the chest, abdomen, and pelvis showed a 13.5 x 7.6 cm enhancing, exophytic mass of the left kidney and innumerable bilateral pulmonary nodules concerning for metastatic malignancy. Cytoreductive nephrectomy was performed in November 2017 and pathology showed clear-cell RCC with sarcomatoid features. Approximately five weeks later, he was initiated on systemic treatment with sunitinib 50 mg daily, six-week cycles with a two-weeks on, one-week off schedule.

Laboratories at baseline showed hemoglobin 9.1 g/dL and MCV 88.1 fL. Imaging with computed tomography after three cycles of sunitinib showed a partial response; however, serial laboratories showed the development of worsening macrocytic anemia with hemoglobin 6.6 g/dL and MCV 106.9 fL. Further laboratory workup showed total bilirubin 2.6 mg/dL, direct bilirubin 0.2 m/dL, lactate dehydrogenase 210 U/L, and haptoglobin 27 mg/dL. Direct antiglobulin testing was negative and iron studies, thyroid function tests, and liver tests were normal. Notably, the patient was found to have significant deficiencies in cobalamin (<146 pg/dL; normal, 213-816) and folate (5.9 ng/dL; normal, > 7) and peripheral smear showed numerous hypersegmented neutrophils. Testing for antibodies against parietal cells and the intrinsic factor was negative. There were no prior values of cobalamin or folate for comparison. On review of history, the patient endorsed a balanced diet with adequate intake of sources of folate and cobalamin, had no history of gastrointestinal surgeries, and denied significant alcohol use. Treatment of nutritional deficiencies was initiated with folic acid one milligram by mouth daily and cobalamin intramuscular 1000 mcg for one dose followed by cobalamin one gram by mouth daily. The patient was transfused with two units of packed red blood cells. Although there was some concern that these findings represented toxicity from sunitinib, given evidence of clinical benefit the decision was made to continue treatment with close monitoring of hematologic parameters.

Repeat laboratories during cycle four of sunitinib showed stable blood counts and normalization of cobalamin (813 pg/dL) and folate (>20 ng/mL) levels, however during cycle five the patient complained of worsening fatigue and exertional dyspnea, and laboratories showed worsening macrocytic anemia with hemoglobin 7.1 g/dL and MCV 108.0 fL. Cobalamin and folate levels remained normal. The patient was transfused with two units packed red blood cells and sunitinib was held.

After four weeks of sunitinib, laboratories showed hemoglobin 9.2 g/dL and MCV 100.5 fL, and the patient reported a marked improvement in energy. Sunitinib was restarted at a reduced dose of 37.5 mg daily on two weeks on and one week off schedule. The patient was also started on a regimen of parenteral cobalamin (1000 mcg subcutaneous daily for seven days, then weekly for four weeks, then monthly) and continued on oral folate. Following one cycle, hemoglobin was relatively stable at 8.6 g/dL and MCV had improved to 93.9 fL. Labs for several cycles thereafter showed stable levels of hemoglobin, folate, and cobalamin. Current treatment plans are to continue sunitinib with close hematological monitoring and ongoing supplementation with oral folate and parenteral cobalamin.

## Discussion

Macrocytosis is a well-documented side effect of sunitinib. Retrospective analyses have shown that macrocytosis develops in a large proportion of patients after approximately three months of treatment, worsens with continued treatment, and resolves with discontinuation of sunitinib [1]. In the largest study, Rini et al. showed that in a cohort of patients treated with sunitinib for at least three months, 41 out of 61 patients (67%) had macrocytosis at some point during their course, with a median increase in MCV of 5.1 fL (*p *< 0.001) at three months. Additionally, among 10 patients in the analysis who had laboratories 2-3 months after discontinuation of sunitinib, MCV decreased by a median of 9.7 pL (*p *= 0.002) [2]. Notably, some studies suggest macrocytosis secondary to treatment with sunitinib could be a marker of progression-free survival in RCC, as has been found with other TKI-associated toxicities such as hypertension and abnormal thyroid function [3,6]. 

Sunitinib-associated macrocytosis has been hypothesized to occur via off-target inhibition of the stem cell growth factor c-Kit, leading to impaired erythrocyte maturation [2]. This conjecture is bolstered by the observation that macrocytosis is seen in 42% of patients treated with imatinib, a potent inhibitor of c-Kit, but is not seen with the VEGFR-targeted TKI sorafenib, which has only weak activity against c-Kit [2-3].

Although sunitinib-associated macrocytosis and anemia may occur secondary to the inhibition of erythrocyte maturation, there have also been, to our knowledge, at least three reports of an association with cobalamin deficiency. In 2007, two small case series were published successively in The New England Journal of Medicine. Gilleson et al. reported on six patients who developed macrocytosis after treatment with sunitinib. All patients had deficiencies in cobalamin and five had reduced levels of holotranscobalamin. Folate was normal in all patients and none developed worsening anemia or symptoms of cobalamin deficiency [5]. Shortly thereafter, Billemont et al. showed that in a cohort of 10 patients receiving sunitinib who developed marked macrocytosis (MCV > 105 fL), mean levels of cobalamin (112 pmol/L; normal level, >133) and folate (4.03 nmol/L; normal level, >6.8) were decreased, and five of 10 patients had anemia (mean hemoglobin 8.84 g/dL) [4]. In a later retrospective study of 27 patients treated with sunitinib, eight patients who developed macrocytosis had levels of cobalamin and folate examined. Four patients were found to have cobalamin deficiency, while none had evidence of folate deficiency. Patients with cobalamin deficiency had a non-significant trend toward higher MCV compared to those with normal levels (104.9 + 4.9 vs 100 + 0.6 fL after three cycles; *p *= 0.2) [3].

Other reports have contradicted these findings. In a retrospective analysis of 21 patients with sunitinib-related macrocytosis, Price et al found cobalamin deficiency in only two of seven patients for whom data were available, a smaller proportion than other studies. Folate deficiency was not found in eight patients [7]. Additionally, the previously mentioned retrospective analysis by Rini et al did not find abnormal levels of cobalamin (*n *= 12; median, 422 pg/mL; normal, 221-700) or folate (*n* = 10; median, 15.4 ng/mL; normal, 2.8-18) [2].

A theoretical mechanism for sunitinib-associated cobalamin deficiency has not been clearly elucidated. In general, cobalamin deficiency can occur via reduced absorption, reduced release from stores, or increased cellular consumption [8]. Gilleson hypothesized that given that there is no known association between TKIs and cobalamin metabolism, impairment of gastrointestinal absorption may be the most likely mechanism. Patients in their series were treated with parenteral cobalamin, and none developed worsening anemia [5]. Our patient had evidence of megaloblastic anemia that persisted despite oral cobalamin replacement and improved with parenteral replacement. Although his cobalamin level did improve with oral therapy, laboratory testing of cobalamin comes with caveats: various conditions including malignancy can lead to inaccurate measurement and tissue stores of cobalamin are only moderately correlated with serum levels [8]. On the other hand, it is known that cobalamin stores in healthy adults are usually large and cobalamin deficiency typically takes years to develop. Given deficiency developed over only a few months in these series, this argues against impaired gut absorption as the sole cause of sunitinib-associated deficiency and we hypothesize that the mechanism may be multifactorial. Further studies to clarify the frequency of sunitinib-associated cobalamin deficiency and determination of its cause would be useful.

## Conclusions

Macrocytosis and anemia are common effects in patients treated with sunitinib and likely occur via inhibition of c-Kit. Data from several small studies suggest that a subset of patients can also develop megaloblastic anemia secondary to cobalamin deficiency. The mechanism for this effect is not clear. Patients with more severe macrocytosis may be more likely to have evidence of cobalamin deficiency. In a small case series, parenteral cobalamin replacement therapy appeared to mitigate these effects. Our patient had evidence of severe, transfusion-dependent megaloblastic anemia which persisted despite oral cobalamin replacement but has improved over a short interval of parenteral replacement. While our patient also had decreased levels of folate, evidence for an association with folate deficiency is less convincing and has been seen in only one small case series. Given good evidence for cobalamin deficiency as a rare side effect of sunitinib, this should be added to materials describing the possible toxicities associated with sunitinib in order to promote physician awareness. Further studies would be useful, and clinicians should consider monitoring cobalamin levels in patients who develop macrocytosis while on treatment with sunitinib.
